# Hyperoxia Inhibits the Growth of Mouse Forebrain Oligodendrocyte Progenitors but Promotes Their Differentiation

**DOI:** 10.1080/17590914.2026.2698260

**Published:** 2026-09-20

**Authors:** Lisamarie Moore, Lauren McLane, Stacey Wahl, Isis M. Ornelas, Teresa L. Wood, Peter Canoll, Steven W. Levison

**Affiliations:** a Rutgers – New Jersey Medical School, Department of Neurology & Neurosciences; b Rutgers –School of Graduate Studies, Newark, NJ, USA; c Rutgers – New Jersey Medical School, Center for Cell Signaling, Newark, NJ, USA; d Departments of Pathology and Cell Biology, Columbia University, NY, USA

**Keywords:** Cell culture, development, mice, myelin, oxygen

## Abstract

Oligodendrocytes facilitate saltatory conduction and they support both neuronal and axonal survival; thus, when they are diseased or damaged, neurological problems ensue. Oligodendrocyte progenitors (OPCs) have been successfully isolated from rodents using magnetic beads, immunopanning and differential adhesion. Whereas rat OPCs are easy to propagate *in vitro*, expanding mouse OPCs in vitro has not been straightforward, which is problematic given that mice have been used to generate the majority of genetically engineered disease models. In this study, we developed and characterized a reproducible, straightforward method to prepare large numbers of nearly homogeneous cultures of mouse OPCs. Using the McCarthy and de Vellis mechanical separation method, we isolated OPCs from a mixture of glial cells and plated them onto fibronectin-coated tissue culture plates in a biochemically defined medium supplemented with fibroblast growth factor-2 (FGF-2) and medium conditioned by B104 neuroblastoma cells. However, when maintained in a standard tissue culture incubator, they proliferated very slowly and ∼ 20% died. By contrast, when they were maintained in an incubator with 2% oxygen they proliferated more rapidly and could be expanded for multiple passages with high viability. After three passages, greater than 99% of these OPCs expressed markers of early OPCs. In medium containing only FGF-2 they progressed to late-stage OPCs. When a medium supplemented with T3 was provided, a large subset of OPCs differentiated into O4+/MBP+ oligodendrocytes with sheet-like membranes. However, between 3 and 6 passages, less than 20% of the OPCs differentiated into MBP+ oligodendrocytes and this was associated with a loss of Sox-10. These studies reveal significant differences between mouse and rat OPCs and a role for oxygen tension in mouse OPC proliferation and differentiation.

## GRAPHICAL ABSTRACT

**Figure:**
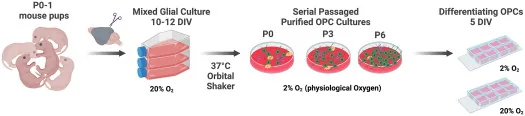

## Introduction

One of the last cell types to develop from the central nervous system (CNS) neuroepithelium is the oligodendrocyte, which becomes the myelinating glial cell of the CNS. These cells play a critical role in facilitating saltatory conduction of neuronal action potentials, as well as supporting axonal survival (Noble & Wolswijk, 1992; Wolswijk & Noble, 1989). Oligodendrocytes are concentrated in the white matter but are also found extensively throughout the brain parenchyma. Most adult OPCs remain as immature proliferative cells, while a subpopulation can differentiate into mature oligodendrocytes to replace dying or dead cells throughout their lifespan (Nishiyama et al., 2009; Trotter et al., 2010).

Oligodendrocyte progenitor cells (OPCs) arise during late embryonic development. They undergo several distinct stages of maturation that can be distinguished using antigens expressed at those specific stages (Gard et al., 1995; Pfeiffer et al., 1993). Platelet-derived growth factor receptor alpha (PDGFRα), a tyrosine kinase receptor, is expressed by early-stage OPCs (Li et al., 2017). OPCs that express PDGFRα generally have a bipolar morphology. These cells also express tetrasialogangliosides that are recognized by the A2B5 monoclonal antibody. A2B5 is found on multiple CNS cell types, but when expressed by an OPC, it identifies it as an early progenitor cell (Eisenbarth et al., 1979; Raff et al., 1983; Schnitzer & Schachner, 1982). Another useful antigen for the early OPC is the nerve/glial antigen 2 (NG2), a chondroitin sulfate proteoglycan (Li et al., 2017; Nishiyama et al., 2009). These markers are helpful in identifying immature OPCs but must be used with care as both can label other neural precursor cell populations. For example, a subset of NG2 and A2B5-expressing cells maintains the capacity to terminally differentiate into both astrocytes and oligodendrocytes (Nishiyama et al., 2009; Zhu et al., 2008, 2011).

As OPCs mature into oligodendrocytes, they begin to express antigens recognized by O4, a monoclonal antibody that binds to the cell-surface pro-oligodendroblast antigen (POA) and sulfatide, which are found on late-stage OPCs and postmitotic oligodendrocytes (Bansal et al., 1992; Sommer & Schachner, 1981). O4 serves as a more reliable marker for OPCs committed to an oligodendroglial fate than A2B5 or NG2 alone (Nishiyama, 1998; Nishiyama et al., 2009; Trotter et al., 2010). O4 expressing cells typically have a multipolar morphology. Finally, mature myelinating oligodendrocytes express myelin-specific antigens such as galactocerebroside and myelin basic protein (MBP). MBP is one of the primary protein constituents of myelin and is regularly used as a marker for mature oligodendrocytes (Dubois-Dalcq et al., 1986; Monge et al., 1986; Trotter et al., 2010).

The isolation and *in vitro* study of OPCs from both the developing and adult CNS has provided critical insights into the properties of these cells and their potential to repair the white matter damage caused by demyelinating diseases such as multiple sclerosis or traumatic injury to the brain and spinal cord. Numerous rodent oligodendroglial cell lines have been developed, such as the rat CG4 cell line (Louis et al., 1992) and the mouse Oli-Neu cell line (Trotter et al., 1993), but the molecular changes that occur as these cells become transformed render them inadequate for many studies intended to provide insights into oligodendrocyte normal function and myelination.

Several methods for isolating rat OPCs from the CNS have been described, such as immunopanning (Barres et al., 1992b; Behar et al., 1988; Gard et al., 1993) fluorescence-activated cell sorting (FACS) and magnetic-activated cell sorting (MACS) by exploiting the cell surface-specific antigens described above (Behar et al., 1988; Dincman et al., 2012; Gard et al., 1995; Horiuchi et al., 2010). However, most of these methods use A2B5 or NG2 selection and, thus, will capture cells that are not committed to the oligodendroglial lineage. Other methods have included differential gradient centrifugation (Althaus et al., 1984; Duncan et al., 1992; Goldman et al., 1986; Vitry et al., 1999), formation of oligospheres (Chen et al., 2007b) and lastly the shaking method based on differential adherent properties of glia (McCarthy & de Vellis, 1980). This latter method permits the separation of rat OPCs from an astroglial bed layer by centripetal forces. The McCarthy and deVellis method has been very successful for isolating rat OPCs; however, when applied to mouse OPCs, this procedure has been relatively unsuccessful. Since most transgenic and knockout studies are carried out in mice, there is a crucial need to develop a simple procedure to produce large quantities of OPCs with high purity from mice.

## Materials and Methods

### Mixed Glial Cell Cultures

Cultures were established from C57BL/6 mouse telencephala, from postnatal day 0 (P0) to day 2 (P2). On average, two litters were combined, yielding 15–20 mice per experiment. Mice were decapitated under sterile conditions and their brains were placed into chilled phosphate-buffered saline (PBS) supplemented to 0.6% d-glucose (Sigma, Cat#G-7528) and 2 mM MgCl_2_ (ThermoFisher Scientific, Waltham, MA, USA, Cat# BP214–500) (a buffer we refer to as PGM) on ice blocks. A block of tissue excised between the olfactory bulbs and the cerebellum was cut in half, and the meninges were carefully removed using fine forceps. These blocks were transferred to fresh PGM and the isolated tissue was gently minced into ∼ 1 mm square pieces with forceps. The tissue was then transferred to conical tubes using a large-bore P1000 tip or a 10 mL pipette, and centrifuged at 200 x g for 10 minutes. The pellet was enzymatically dissociated using a pH-balanced solution containing 0.125% trypsin (Sigma, Cat#T4549) and DNase I (0.02 mg/mL) (Sigma-Aldrich, Cat#D5025) in a volume of 10 mL of minimum essential media (MEM) (CellGro, Cat#50-010). The tissue was digested for 10 minutes at 37 °C with manual agitation performed several times during incubation. An equal volume of medium supplemented to 10% fetal bovine serum (FBS) (Atlantic Biologicals, Cat #S11150) was added to quench the trypsin. The mixture was gently triturated for several cycles using a 10 mL pipette, adding additional media during later cycles. The cell suspension was passed through a 100 µm cell strainer to eliminate clumped cells from the final mixture. The cells were centrifuged at 400 x g for 8 minutes. Finally, the supernatant was removed and passed through a 40 µm cell strainer. Viable cells were determined by Trypan blue exclusion and plated at 2 × 10^5^ cells/cm^2^ in MEM-10C media (MEM medium containing 10% (v/v) pre-screened^1^ FBS, 200 mM L-Alanyl-L-Glutamine (Corning-CellGro, Cat #25-015-CI), 1% Penicillin-Streptomycin solution (Corning-CellGro, Cat #30-002-CI), 15 mM HEPES (Gibco Cat#15630-080) and 0.6% d-glucose. Cells were cultured in twist-cap Falcon tissue culture straight-neck T75 flasks (Becton Dickinson, Cat#3024) for 10–12 days, with the first medium change occurring 4 days after initial plating and then every other day thereafter. All animal work was performed according to approved Rutgers NJMS Institutional Animal Care and Use Committee protocols #10012 and #999900840.

### Isolation and Purification of Mouse Oligodendrocyte Progenitors

Once the bed layer of the mixed glial cultures was confluent (∼9 days), the mouse OPCs were isolated using the well-established mechanical separation technique (McCarthy & de Vellis, 1980) with modifications to improve cell survival as described previously (Young & Levison, 1997). The twist-caps on the T75 Falcon flasks were tightened, and then the flasks were subjected to centripetal shaking at 260 rpm on a Labline #3525 orbital shaker (1” orbit diameter) for 1.5 hours at 37 °C to remove lightly adhered microglia. Flasks were gently agitated manually to remove any remaining adherent microglia, followed by supernatant removal. Cells were rinsed twice with unsupplemented MEM and then returned to the incubator in MEM-10C for at least 1 hour to equilibrate with 5% CO_2_ at 37 °C. The twist caps were tightened once more, and the cells were shaken overnight at 260 rpm for 18 hours to remove the attached OPCs, leaving the more adherent astrocytes behind. The flasks were gently agitated manually to further dislodge any remaining OPCs. The supernatant was removed and very slowly passed through a 20 µm nitex mesh (Component Supply Co, Sparta, TN, Cat# B0013HKT14) affixed to a sterile beaker to remove any clumps of astrocytes that may have been dislodged. Unsupplemented MEM was added to the flask twice to gather any remaining OPCs. The cells were then transferred to a conical tube and centrifuged at 400 x g for 5 minutes. Cells were resuspended in B27 growth medium [DMEM: F12-HEPES (Gibco, Cat# 21331-020) supplemented to 1X (v/v) B27 supplement (Gibco, Cat# 17504044), 1% (v/v) Penicillin-Streptomycin solution (ThermoFisher, Cat#15140122) and 0.5% (v/v) prescreened FBS. The cells were plated onto a 100 mm bacteriological plastic dish (Falcon Cat. #1029) for 30 minutes at 37 °C to remove contaminating microglia (Behar et al., 1988). Bacteriological plastic dishes were rinsed twice with B27 growth medium to collect the nonadherent OPCs while leaving behind any attached microglia. The supernatant was centrifuged at 400 × g for 5 minutes. Viable cells were counted and resuspended in proliferation media N2S [B27 growth medium supplemented with 30% B104 neuroblastoma conditioned medium (B104 CM)(Young & Levison, 1997), or 10 ng/mL recombinant rat platelet-derived growth factor (PDGF-AA) (R&D Systems, Cat# 1055-AA-050) and 10 ng/mL FGF-2 (Peprotech, Cat#100-18C or Alomone Labs, Cat#F-170) with 1 ng/mL heparin sodium (Sigma-Aldrich Cat#H3149). OPCs were plated onto 100 mm tissue culture plates that had been previously coated with 0.5 µg/cm^2^ fibronectin MW > 440kD (BD Biosciences Cat#354008) for 2 hours @ 37 °C. The OPCs were seeded at 3x10^4^ cells/cm^2^ and maintained in either 2% O_2_, 5% CO_2_, 93% N_2_ or 20% O_2_, 5% CO_2_, 75% N_2_ @ 37 °C. Half of the medium was changed every other day until the cells were approximately 80% confluent.

### Amplifying and Differentiating Mouse Oligodendrocyte Progenitors

OPCs were gently removed from the fibronectin-coated plates using a freshly prepared solution containing 44 U/mL papain (Worthington, Cat# PAP) and DNase I in MEM with 20 mM Hepes, 5 mM EDTA (Sigma-Aldrich Cat#E1644-250G), and 14 mM L-cysteine (Sigma Cat#C7352-25G). The papain was activated by pre-incubating this solution in a C0_2_ culture incubator for 30 minutes and then vortexing well before use. The solution was swirled over the cells and then aspirated to leave a thin film of solution. The cells were returned to the incubator for 7 minutes. The cells were detached by pipetting 5 mL of B27 growth medium and the cells were collected by centrifugation at 400 x g for 8 minutes. The pellet was resuspended, and viable cells were plated in N2S onto fibronectin-coated flasks at no less than 2.5x10^4^ cells/cm^2^. Again, half of the media was changed every other day until the cells reached 80% confluence and were ready to be passaged. To differentiate the OPCs, the mitogens were removed from the media and the cells were placed into B27 growth medium supplemented with 30 nM triiodothyronine (T3) (Sigma Cat#T-2877) for 5 days in either 2% O_2_, 5% CO_2_, 93% N_2_ or 5% CO_2_ in room air (∼20% O_2_), @ 37 °C.

Rat OPCs were generated and propagated as described previously (Young & Levison, 1997).

### Immunofluorescence

Cells were replated onto fibronectin-coated chamber slides (LabTek Cat# 177402) and maintained up to 5 days in N2S (proliferation media) or B27 growth medium supplemented with T3 (differentiation media). To identify astrocytes, microglia, and OPCs, cells were stained live for mouse anti-O4 (1:4, in-house generated supernatant), mouse anti-A2B5 (1:4, in-house supernatant), rabbit anti-NG2 Chondroitin Sulfate Proteoglycan (1:50 Millipore, Cat#AB5320, MAB5384) and rat anti-PDGFRα (1:100 Cell Signaling, Cat#3164) for 1 hour at room temperature, then rinsed and fixed with 4% paraformaldehyde (PFA), followed by 100% ice chilled methanol. The cells were then stained for Iba-1 (Wako Chemicals, Cat # 019–19741) or for GFAP (GF2.2 in-house generated supernatant). After multiple rinses, the cells were incubated with appropriate fluorochrome-conjugated secondary antibodies for 1 hour at room temperature. The cells were then blocked, permeabilized and incubated with mouse or rabbit anti-Olig2 (1:100, Millipore Cat#387M-15 or Cat# AB9610), Ox42 (1:50, Serotec, Cat#MCA275G), rabbit anti-GFAP (1:500, Dako Cat#Z0334), goat anti-Sox10 (1:50, R&D Systems, Cat#AF2864) and rabbit anti-Ki67 (1:750, Vector Cat#VP-K451). The cells were washed and further incubated with secondary antibodies. Some cells were counterstained with 1 µg/ml DAPI (Sigma, Cat# D9542) for 5 minutes. For differentiation studies, cells were stained with mouse anti-MBP (1:250, Covance, Cat#SMI-99P). All secondary antibody combinations were carefully examined to ensure no bleed-through between fluorescent dyes or cross-reactivity between secondary antibodies. No signal above background was obtained when the primary antibodies were replaced with pre-immune sera. All secondary antibodies were purchased from Jackson ImmunoResearch.

Stained cells were washed thoroughly and mounted with Fluorogel (Electron Microscopy Sciences, Cat# 17985-10) or ProLong Gold Antifade Mountant with DAPI (Invitrogen, Cat# P36935) and allowed to dry overnight. Immunoreactive cells were visualized using an Olympus Provis AX70 microscope (Center Valley, PA, USA) and images of stained cells were collected using a Q-imaging CCD camera (Surrey, BC, Canada) interfaced with IP Lab scientific imaging software (Scanalytics, Rockville, MD, USA). Labeled cells in at least 4–5 random (nonadjacent) fields per well were counted under 20X or 40X objective and a total of 4 wells per independent group were evaluated with at least 100 cells counted based on DAPI staining to evaluate the purity of the isolation and amplification technique described above.

### Surface Marker Analysis by Flow Cytometry

Cell surface expression was assessed by flow cytometry using the following protocol. OPCs were gently removed from the tissue culture plates with 0.2 Wünsch units (WU)/mL of Liberase DH (Roche) and I in PGM at 37 °C for 5 minutes. An equal volume of PGM plus DNase I without Liberase was added and the plates were vigorously agitated. Detached cells were collected by centrifugation at 400 x g for 5 minutes. Cells were resuspended in 10 mL of PGB [PGM supplemented with 2 mg/mL fraction V of Bovine Serum Albumin (Sigma, cat # A-6003) and centrifuged for 5 minutes at 400 x g. Cells were dissociated by repeated trituration, and viable cells were counted using a hemocytometer. Cells were segregated into 1x106 cells per experimental group (stained, isotype control, live/dead and unstained). Prior to staining, cells were resuspended in 50 µL of FcR block in PGB (1:50, BD Pharmigen, Cat # 553141). All staining was performed in 650 µL tubes using a total volume of 150 µL. For surface marker analysis, cells were incubated with unconjugated mouse O4 for 25 minutes at 4 °C. Cells were washed and then incubated with anti-mouse IgM-PerCP-eFluor 710 (1:200; eBioscience, Catalog #46-5790-82) in 1:4 rat serum for 25 minutes on ice. Cells were blocked with nonspecific IgMs to bind all available epitopes on the secondary, washed, and then incubated with unconjugated rabbit anti-NG2 and mouse anti-Glutamate Aspartate Transporter (GLAST) (1:11; ACSA-1, Miltenyi, Cat #130-118-984). After washing with PGB, cells were incubated with appropriate secondaries [goat anti-rabbit IgG Alexa Fluor 700 (1:100; Molecular Probes, Cat # 2129003) and Streptavidin-APC-eFluor 780 (1:160; eBioscience, Cat#47-4317-82) along with directly conjugated antibodies CD133-APC (1:50; 13A4, eBioscience, Cat# 17-1331-81), Lewis-X-V450 (1:20; MMA, BD Bioscience, Cat#561561), CD140a-Pe (1:400; APA5, Biolegend, Cat #135906) and 1:3200 DAPI solution with 1:4 goat serum for 25 minutes. To restrict our analysis to living cells, we used a live/dead cell exclusion technique that identified DAPI-positive cells as dead and DAPI-negative cells as alive. Cells were washed with PGB by centrifugation at 500 x g and lightly fixed with 1% ultrapure formaldehyde in PBS w/o Mg2+ and Ca2+. Whenever possible, the cells were analyzed immediately after completing the staining protocol. All sample data were collected on the BD LSR II (BD Biosciences Immunocytometry Systems) with excitation lasers at 350, 407, 488, and 633 nm and bandpass filters for APC (660/20), V450 (440/40), Pe (575/26), Alexa 488 (530/30), Alexa700 (710/20), APC-eFluor 780 (780/60), PerCp-eFluor 710 (695/40) and DAPI (440/40). Gates were set based on matching isotype controls from the same manufacturer as the purchased primary antibodies. Afterward, the acquired data were analyzed by FlowJo software (Tree Star Inc., Ashland, OR).

## In Situ End-Labeling (ISEL)

Cells were dehydrated and rehydrated in ethanol and water, and then incubated with a 10 µM dNTP mix containing Digoxigenin-dUTP (Boehringer Mannheim, Indianapolis, IN, Cat. # 1277065) and 20 U/mL Klenow fragment (Boehringer Mannheim, Cat #1008412) at room temperature for 2 hours. Digoxigenin-labeled nucleotides were detected using Sheep anti-digoxigenin-rhodamine (Boehringer Mannheim, 1:75), which was incubated overnight at 4 °C. Cells were mounted using Fluorogel with DAPI (ThermoFisher, Cat#50-246-93). Images were collected using an Olympus AX70 fluorescence microscope as described above. A particle counting script in FIJI was used to determine the total number of cells and the percentage that were ISEL labeled.

### Statistical Analyses

At least three independent experiments were performed for all studies. Statistical analyses were performed using GraphPad Prism 10 software (San Diego, CA). Sample size tests (power analyses) were performed to estimate the required group sizes for each experiment based on pilot studies (G*Power; University of Düsseldorf). For experiments with continuous variables, results were analyzed for statistical significance using a two-tailed, unpaired Student’s t-test. The means across more than two groups were compared using a One-Way ANOVA with Tukey’s correction. Comparisons were interpreted as significant when *p* < 0.05. Normality (Gaussian) tests (Kolmogorov-Smirnov) were performed before tests that assumed normal distributions. Sphericity tests were performed for all ANOVAs. Outliers were identified and removed from the analyses using the ROUT method (Q = 1%) in GraphPad Prism 10.

## Results

### Mouse OPCs Are Readily Isolated from Mixed Glial Cell Cultures Prepared from Neonatal Forebrain Cultures by Mechanical Shaking

**Figure 1.**
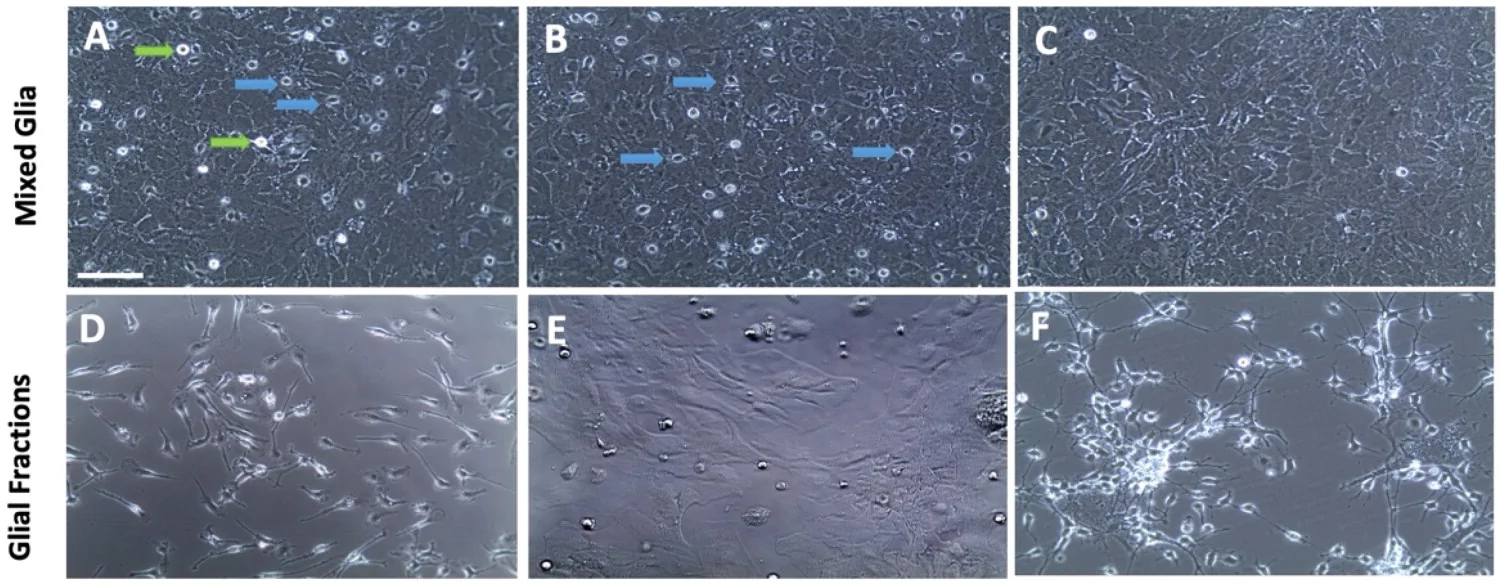
Separation of OPCs from neonatal mouse forebrain mixed glial cell cultures. Mixed glial cell cultures were prepared by dissociating P0 -P2 mouse telencephala and then culturing the cells in 10% serum containing medium in a standard tissue culture incubator (A). At confluency, phase bright cells atop the bed-layer were predominantly microglia (ex. green arrows) whereas the phase dark cells atop the bed-layer were predominantly OPCs (ex. blue arrows). Flasks were shaken at 260 rpm for 1 h to remove microglia and other loosely attached cells and then shaken a second time at 260 rpm for 18 hours to remove OPCs. Representative phase contrast images of the cultures after the first mechanical shake (B) and the second shake (C). Representative phase contrast images of the non-adherent cells obtained from the first mechanical shake (D), the adherent bedlayer after the second shake (E) and the non-adherent cells obtained after the second shake (F). Scale bar represents 100 µm.

Mixed glial cell cultures were prepared from cells dissociated from the telencephala of P0-P2 C57BL/6 mice and plated onto T75 flasks in culture medium containing 10% FBS. These cultures were maintained *in vitro* in standard tissue culture incubators until they reached confluency, which typically required approximately 10–12 days (Figure 1A). At confluency, the cultures consisted of phase-bright microglia (Figure 1A, green arrows) and phase-dark OPCs (Figure 1A, blue arrows) on top of a bed layer of flat, adherent astrocytes (with some fibroblasts). Cultures enriched in OPCs were produced using centripetal forces as described initially by McCarthy and deVellis (McCarthy & de Vellis, 1980). The mixed cultures were placed on a rotary shaker for 1 hour at 260 rpm to remove loosely attached microglia (Figure 1B), followed by an 18-hour shake to remove the majority of the OPCs (Figure 1C).

The microglia enriched fraction was comprised of predominately elongated amoeboid shaped cells (Figure 1D). There were a few OPCs and astrocytes detected in this fraction. The bed layer was comprised mainly of glial fibrillary acidic protein (GFAP)+ astrocytes, with another smaller population of cells that were later identified as α-smooth muscle actin + fibroblasts (Figure 1E). The OPC-containing fraction was comprised of bipolar and tripolar cells, with some contaminating microglia and astrocytes (Figure 1F). These enriched OPCs were passaged as described below to produce a more homogeneous cell population.

### Large Quantities of Mouse OPCs Are Obtained in Vitro When Cells Are Cultured in Media Supplemented with B104 and FGF2 in Physiological Oxygen (2% O_2_)

**Figure 2.**
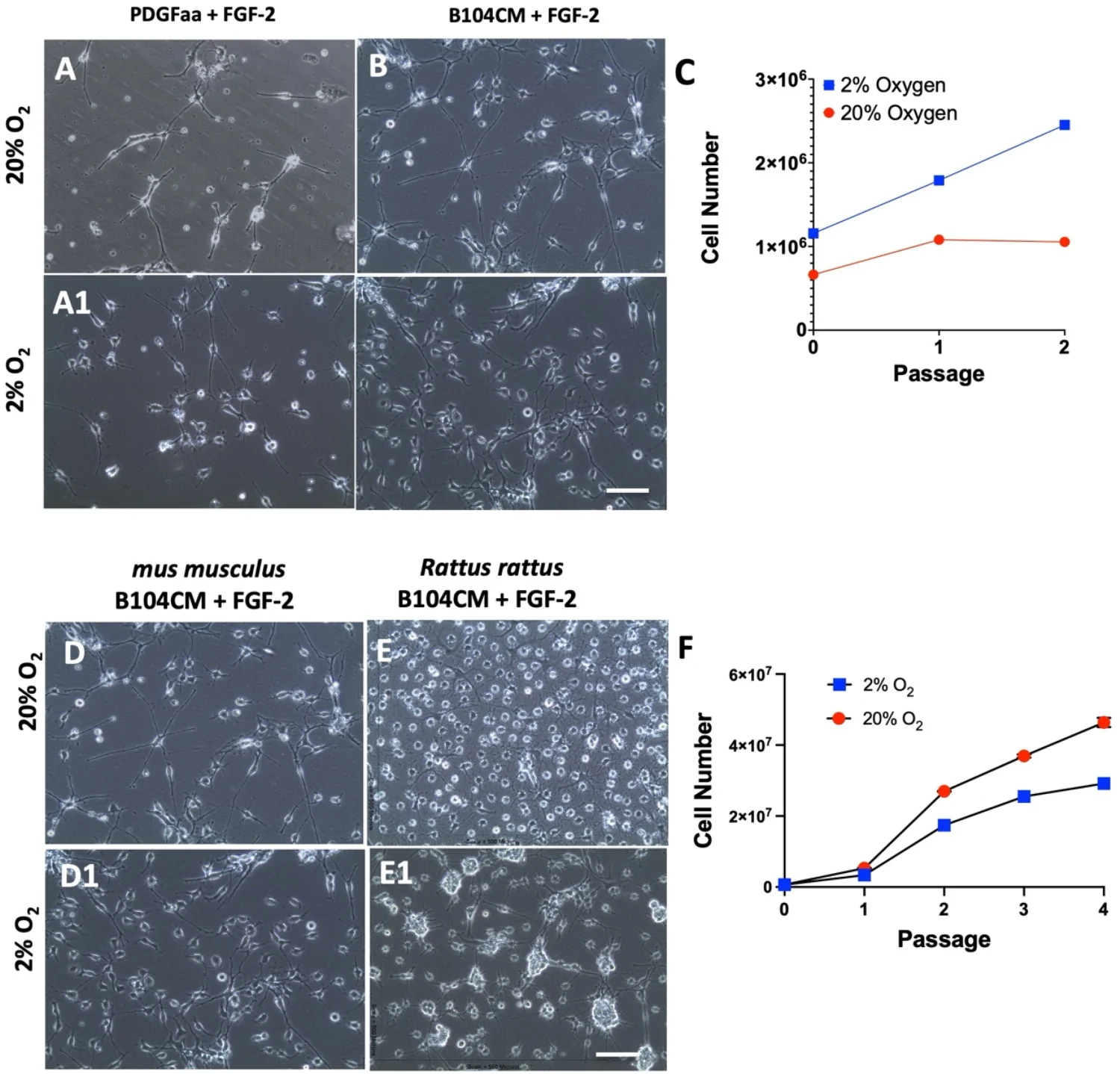
Mouse OPC growth is accelerated when cultured in medium supplemented with B104CM and FGF-2 and maintained under physiological oxygen conditions. The enriched OPC fraction from neonatal C57Blk/6 mouse mixed glial cell cultures was plated onto fibronectin coated tissue culture plates at 3x10^4^cells/cm^2^ and cultured in PDGF-AA+FGF-2 or B104CM + FGF-2 and propagated in 20% oxygen (A, B) or 2% oxygen (A1, B1) for 5 days. The number of cells obtained when grown in B104CM + FGF2 in either 2% or 20% oxygen over 4 serial passages across 35 days is depicted (C). The enriched OPC fractions from mixed mouse glial cell cultures plated onto fibronectin (D) and from mixed rat glial cell cultures plated onto poly-d-lysine (E) and cultured in B104CM + FGF2 in 20% oxygen (D, E) or 2% oxygen (D1, E1) for 2–4 serial passages. The number of rat OPCs plated onto either fibronectin or poly-d-lysine obtained at each passage was counted and plotted on a growth curve (F). Data in C and F are representative of at least 3 independent experiments. Scale bars represent 50 µm.

Initially, the enriched OPC fraction was grown in a medium supplemented with 10 ng/mL PDGF-AA + 10 ng/mL FGF-2 and the cells were plated onto poly-d-lysine (PDL) coated plates as this method has been used effectively to amplify rat OPCs (Bögler et al., 1990; Noble et al., 1988; Raff et al., 1988; Richardson et al., 1988). Under these growth conditions, the OPCs developed elongated processes but they were so firmly affixed to the culture plates that they could not be efficiently passaged (Figure 2A). Therefore, we modified the culture medium, supplementing it with B104 neuroblastoma condition media (B104CM) as this medium had been previously reported to support rat OPC growth (Young & Levison, 1997). B104CM has been reported to contain a high molecular weight, heparin-binding polypeptide growth factor that stimulates the proliferation and survival of rat OPCs (Bottenstein et al., 1988; Hunter & Bottenstein, 1989, 1991). We then tested a variety of enzymes to lift the cells off the culture dish. Although both Accutase and papain can effectively detach rat OPCs from a PDL-coated surface, they were ineffective for the mouse OPCs. As studies have shown that fibronectin promotes the proliferation of OPCs whereas growth on poly-d-Lysine enhances their differentation (Lafrenaye & Fuss, 2010; Ping et al., 2022; Qin et al., 2017), we hypothesized that f the OPCs had more extensive processes then they would be better adhered to the culture dish and more difficult to detach. Therefore, we replaced the culture substratum with fibronectin and with these modifications to the protocol, the cells proliferated and were easily detached using papain; however, they grew very slowly, taking ∼1 month to double in number (Figure 2B).

It was reported that both human and mouse neural progenitors grew better when maintained under physiologically oxygenated medium (Chen et al., 2007a; Pistollato et al., 2007). Therefore, we evaluated OPC growth when cultured in a humidified 3-gas incubator containing 2% oxygen, 5% carbon dioxide, and 97% nitrogen. Cells were cultured in medium containing either 10 ng/mL PDGF-AA + 10 ng/mL FGF-2 (Figure 2A, 2A_1_) or 30% B104CM + 10 ng/mL FGF-2 (Figure 2B, 2B_1_). OPCs grew at a significantly faster rate when cultured in B104CM + FGF-2 in 2% oxygen, and they could be passaged every 4 days compared to cells maintained in the same medium but placed in a 20% oxygen incubator (Figure 2C). Cells maintained in 20% oxygen did expand if left alone for an extended period; however, their numbers never reached the cell numbers obtained in the 2% oxygen incubator. Growth in 2% oxygen significantly increased the overall yield of cells obtained such that by passage three, 4x10^6^ cells were obtained. Cells maintained in 20% oxygen took another passage over 10 days to reach this number. Since the OPCs could not be expanded when maintained in 20% oxygen, we suspected that they might be dying; therefore, cultures were evaluated at 24 and 96 hours using in situ end labeling to assess apoptotic cell death. There were over twice as many apoptotic cells at 96 h in the 20% oxygen condition compared to the 2% oxygen condition (18.7 ± 5% vs. 7.2 ± 3%, *p* < 0.05 by Student’s t-test). There was no significant difference in the percentage of ISEL+ cells at the 24 h time point.

OPC homogeneity increased as the cells were serially passaged in 2% oxygen. Across three independent experiments, the OPC fraction, after three serial passages, achieved 96.48% ± 3.6% A2B5+ positive bipolar immature OPCs with a small percentage of contaminating GFAP+ astrocytes (3.60%±3.4) and a tiny percentage of contaminating Ox-42+ microglia (0.02%±0.003).

In contrast to the mouse OPCs that grew better in the 2% oxygen (compare Figure 2D to 2D1), rat OPCs were insensitive to oxygen. In fact, there was a trend toward better growth in the 20% oxygen incubator compared to the 2% oxygen incubator (compare Figure 2E to Figure 2E1). When rat OPCs were grown in the 2% incubator, they tended to aggregate into spheroids (Figure 2E1).

### OPCs Are Predominantly A2B5+ and Ki67+ When Grown in B104 + FGF2 in Physiological Oxygen

**Figure 3.**
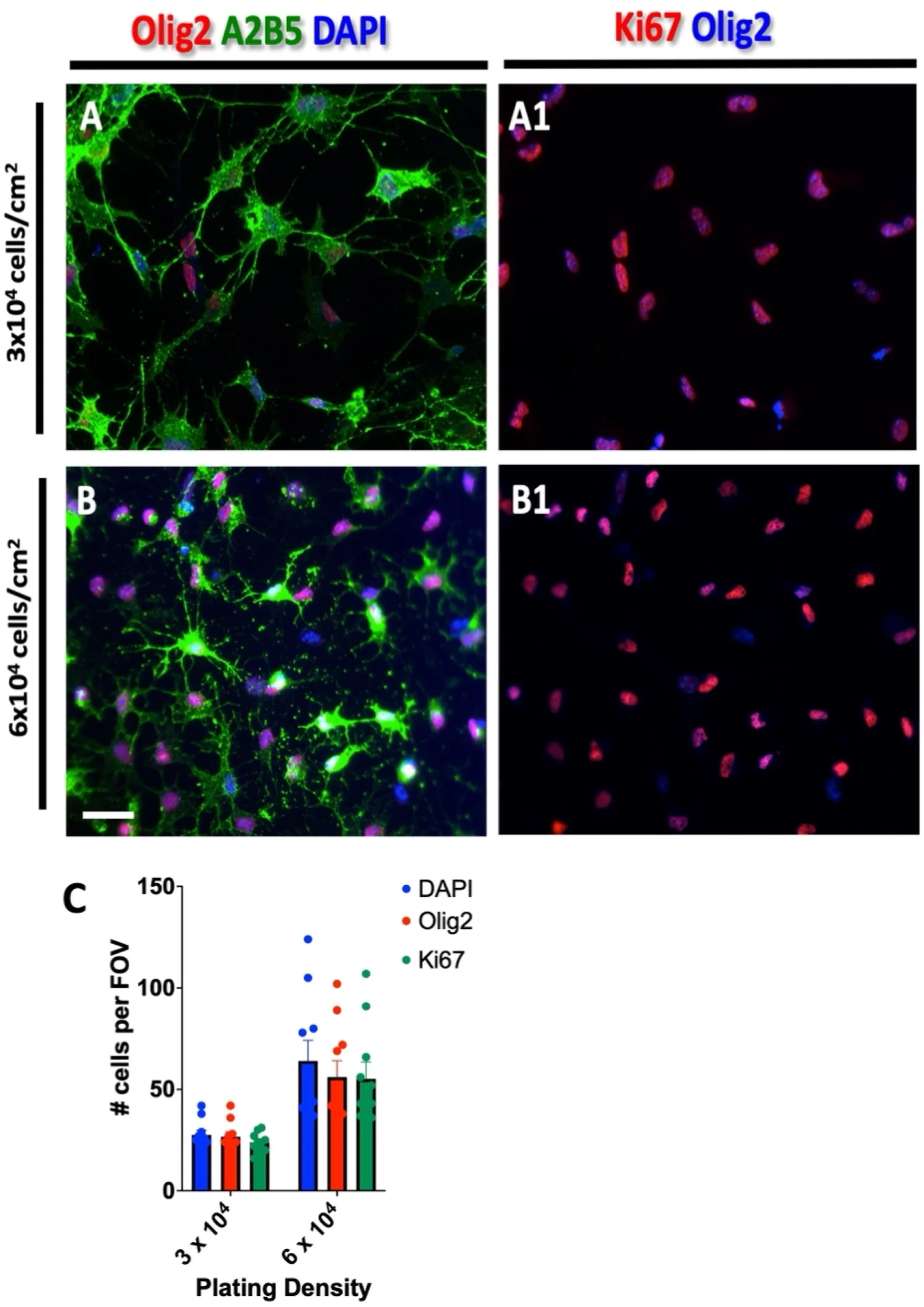
OPCs are A2B5± and Ki67± at both low and high density when grown in B104CM ± FGF-2 in 2% oxygen. After the first passage, OPCs were plated onto fibronectin coated chamber slides at 3x10^4^cells/cm^2^ (25% confluency) (A) and 6x10^4^cells/cm^2^ (50% confluency) (B) and maintained for 4 days in medium supplemented with B104CM + FGF2 in 3 gas incubator with 2% oxygen. Cells were stained for A2B5-green, Olig2-red, and DAPI-blue (A, B) or Olig2-blue and Ki67-red (A1, B1). The number of DAPI+, Olig2+ and Ki67+ cells per field of view (FOV) was quantified (C). Scale bar represents 50 µm. Data represent means ± SEM from three independent experiments.

Next we assessed the effects of cell density on OPC proliferation. OPCs were passaged twice and then plated onto fibronectin-coated chamber slides at 3x10^4^ cells/cm^2^ (approximately 25% confluency) (Figure 3A) and at 6 x 10^4^ cells/cm^2^ (approximately 50% confluency) (Figure 3B). The cells were maintained for 4 days in a 3-gas incubator containing 2% oxygen and then stained for markers of the oligodendrocytic lineage (A2B5 and Olig2). OPCs maintained their early progenitor phenotype with the majority of cells expressing A2B5. The mitotic index, measured as the fraction of Ki67+ cells, was not significantly different between the two conditions and was almost equivalent to the number of Olig2+ cells (Figure 3). For subsequent experiments, the OPCs were plated at 3 × 10^4^ cells/cm^2^ to reduce the passaging frequency.

### Mitogens Have Differential Effects on the Phenotype of Cultured OPCs

**Figure 4.**
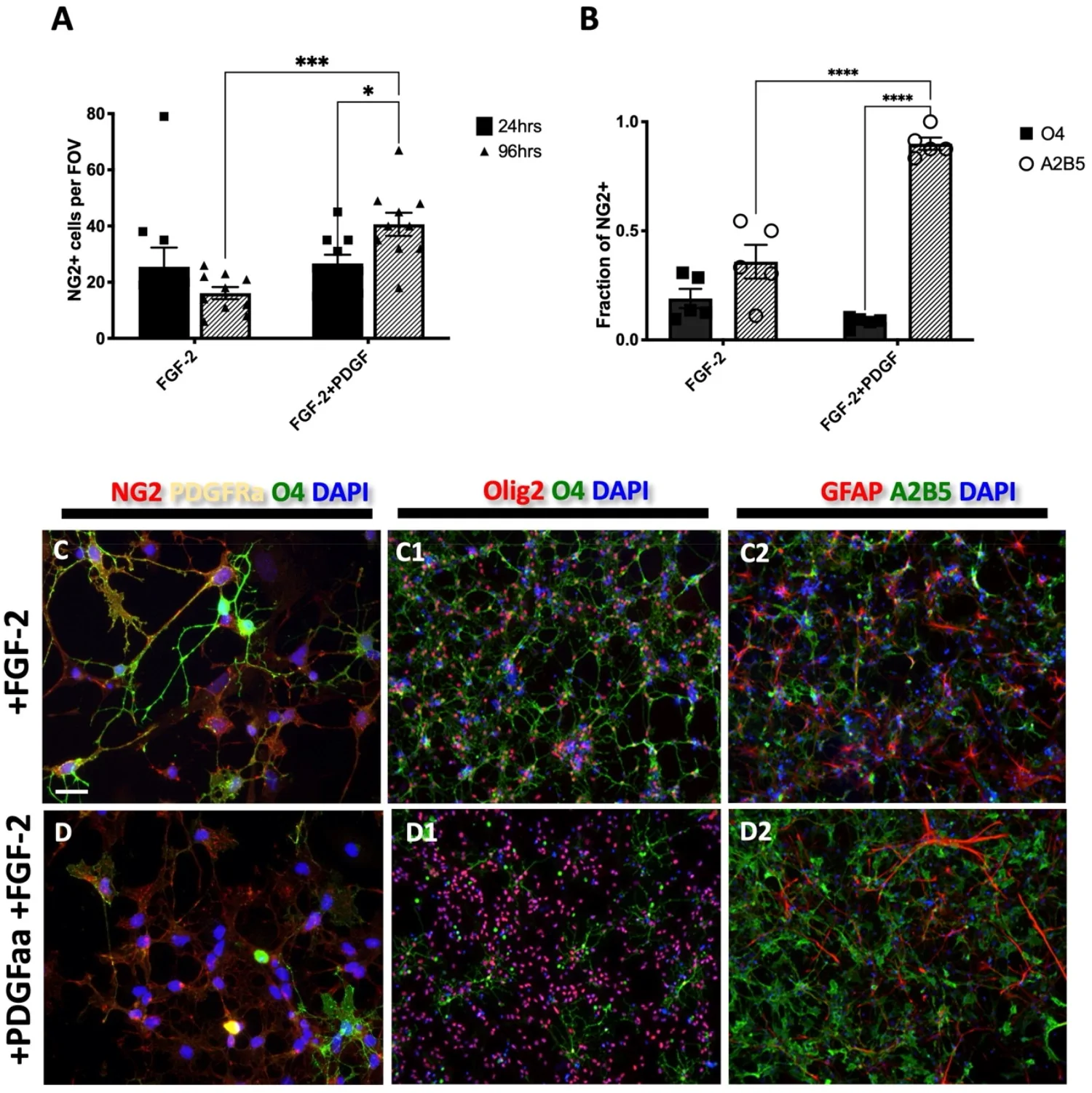
Differential effects of mitogen combinations on the phenotype of cultured OPCs. OPCs were plated onto fibronectin coated chamber slides at 3x10^4^cells/cm^2^ in media supplemented with FGF2 or PDGF-AA+FGF-2 and maintained in 2% oxygen. The number of NG2+ cells was counted at 24 and 96 hours after initial plating (A). The ratio of NG2+ cells that were positive for A2B5 or O4 was quantified at 96 hours after plating (B). Data represent means ± SEM from 3 independent experiments. *p < 0.05, ***p < 0.005, ****p < 0.001 by 2-way ANOVA followed by Tukey’s posthoc test. Cells cultured in FGF-2 or PDGF-AA+FGF-2 were stained with antibodies against NG2 (red), PDGFRα (yellow), O4 (green) and DAPI (blue) (C, D). Cells were stained for O4 (green), Olig2 (red), DAPI (blue) (C1, D1) or GFAP (red), A2B5 (green) and DAPI (blue) after 4 days in culture (C2, D2). Scale bar represents 20 µm (C, D) and 200 µm (C1, C2, D1, D2).

As OPCs mature, they lose A2B5 expression and acquire markers associated with later-stage progenitors, such as O4. To determine whether the combination of PDGF and FGF-2 would prevent mouse OPCs from maturing the cells were cultured in PDGF-AA+FGF-2 or FGF-2 alone for 96 hours after the second shake. At 24 hours, the number of NG2+ cells was equivalent in both conditions; after 96 hours, the number of cells in the PDGF-AA+FGF-2 condition had increased significantly. In contrast, the number of cells cultured with FGF-2 alone did not increase dramatically from the number initially plated (Figure 4A). Similar to rat OPCs cultured in PDGF-AA+FGF-2, most of the cells were A2B5+ (90% ± 0.03), 8% ± 0.01 expressed O4 and there was only a small fraction (2%) that were GFAP+ (Figure 4B, D, D1 D2). Of those cells cultured in FGF-2 alone, a greater percentage started to express O4 (19% ± 0.04) (Figure 4B, 4C, C1, C2). A large fraction of the cells maintained in FGF-2 alone were negative for both A2B5 and O4 (∼40%). These cells stained for Olig2 (Figure 4C1) and unexpectedly GFAP (Figure 4C2).

### OPCs Progress through the Oligodendrocytic Lineage over Time When Grown in B104 + FGF-2 and 2% O_2_

**Figure 5.**
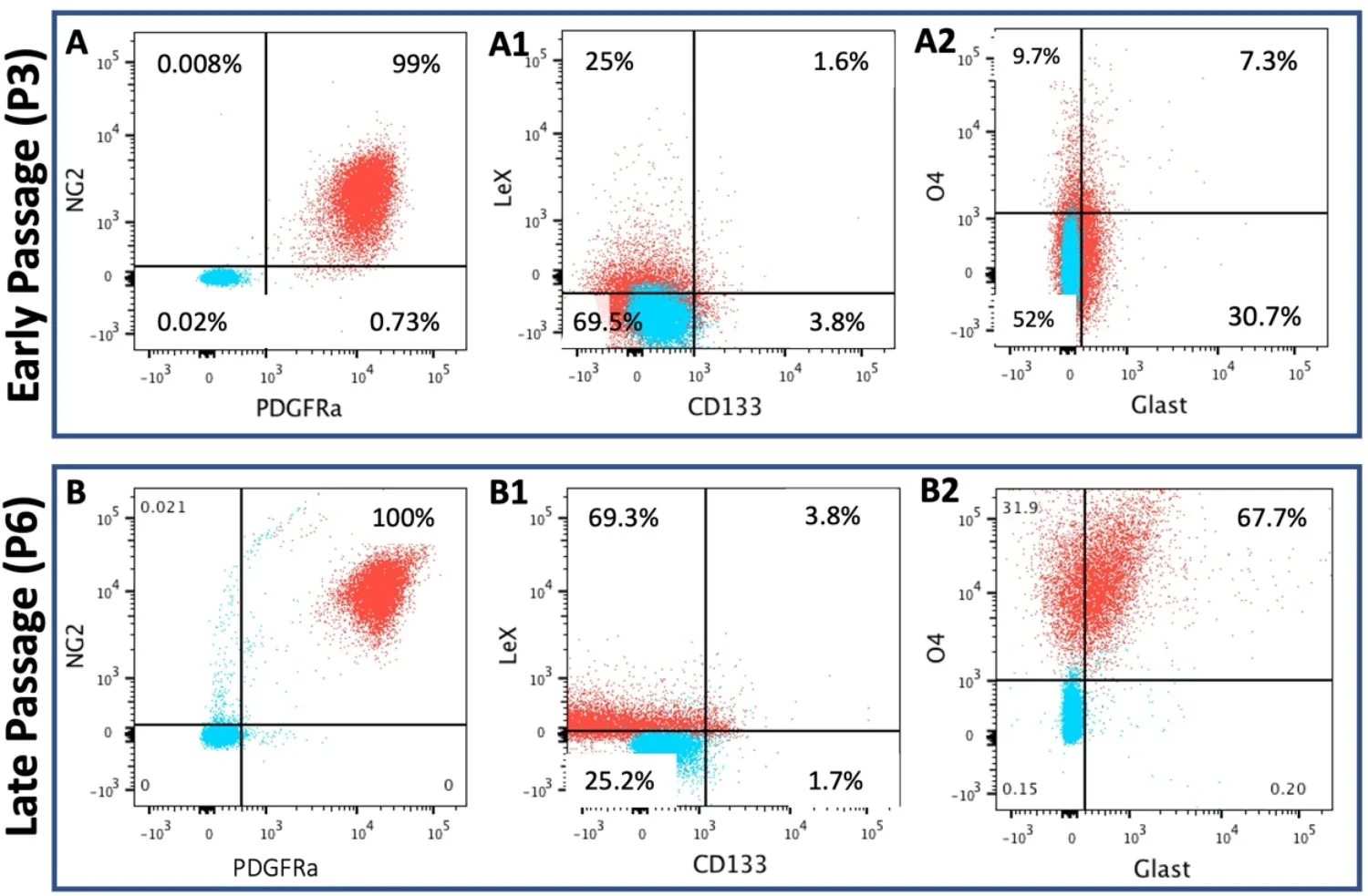
OPCs acquire late progenitor cell markers with time in culture_._ OPCs were grown in B104CM + FGF-2 in a 3-gas incubator with 2% oxygen, passaged, stained and then analyzed live by flow cytometry at passage 3 (A-A2) and passage 6 (B-B2) using a flow panel comprised of CD133/LeX/NG2/CD140a/O4/GLAST. 50, 000 events were analyzed per group. Forward and side scatter were used to exclude debris and DAPI exclusion was used to define live cells. Blue dots represent isotype controls for background and gating while red dots represent the stained events. Data are representative of two independent experiments.

Whereas most immunofluorescence studies are limited to analyzing four fluorescent channels, flow cytometry has been designed to analyze upwards of 13 markers simultaneously. We have found that at least four fluorophores are required to distinguish stem cells from multipotential progenitors (Velloso et al., 2022). Thus, we used flow cytometry to analyze the expression of seven surface markers in these OPC-enriched cultures (Table 1). The markers chosen assessed several stages of OPC maturation, as well as neural stem cells, bipotent and multipotent progenitors. Ninety-nine percent of the cells propagated in B104CM and FGF-2 for 3–6 passages expressed NG2 and PDGFRα (Figure 5A, B). None expressed the phenotype of a neural stem cell marker CD133+/LeX+ (Figure 5A1, B1). However, whereas only 17% of the cells expressed O4 at passage three, expression of O4 increased to 98% by passage 6 (Figure 5A2, B2). Initially only a few of the OPCs expressed GLAST when cultured in B104CM + FGF-2 (Figure 5A2, B2); by passage six, unexpectedly, 68% of the OPCs expressed GLAST.

**Table 1.** Antibodies used to characterize the OPCs.

Antibody	Antigen and/or AKA	Targets
CD133	Prominin-1	Stem cells and Ependymal cells
CD15	Lewis-X, LeX, SSEA-1	Neural precursors, some immature astrocytes
NG2	Chondroitin sulfate proteoglycan	Multipotential, bipotential and unipotential progenitors
CD140a	PDGFRα	Multipotential, bipotential and unipotential progenitors
O4	Sulfatide, POA	Late OPCs
A2B5	Complex Gangliosides	Early OPCs and immature neural precursors
Glast	Glutamate Transporter, EEAT1	NSCs, astrocytes and cerebellar oligodendrocytes

### OPCs Can Differentiate into Mature Myelinating Oligodendrocytes in Vitro When Treated with T3, CNTF and TGFβ1

**Figure 6.**
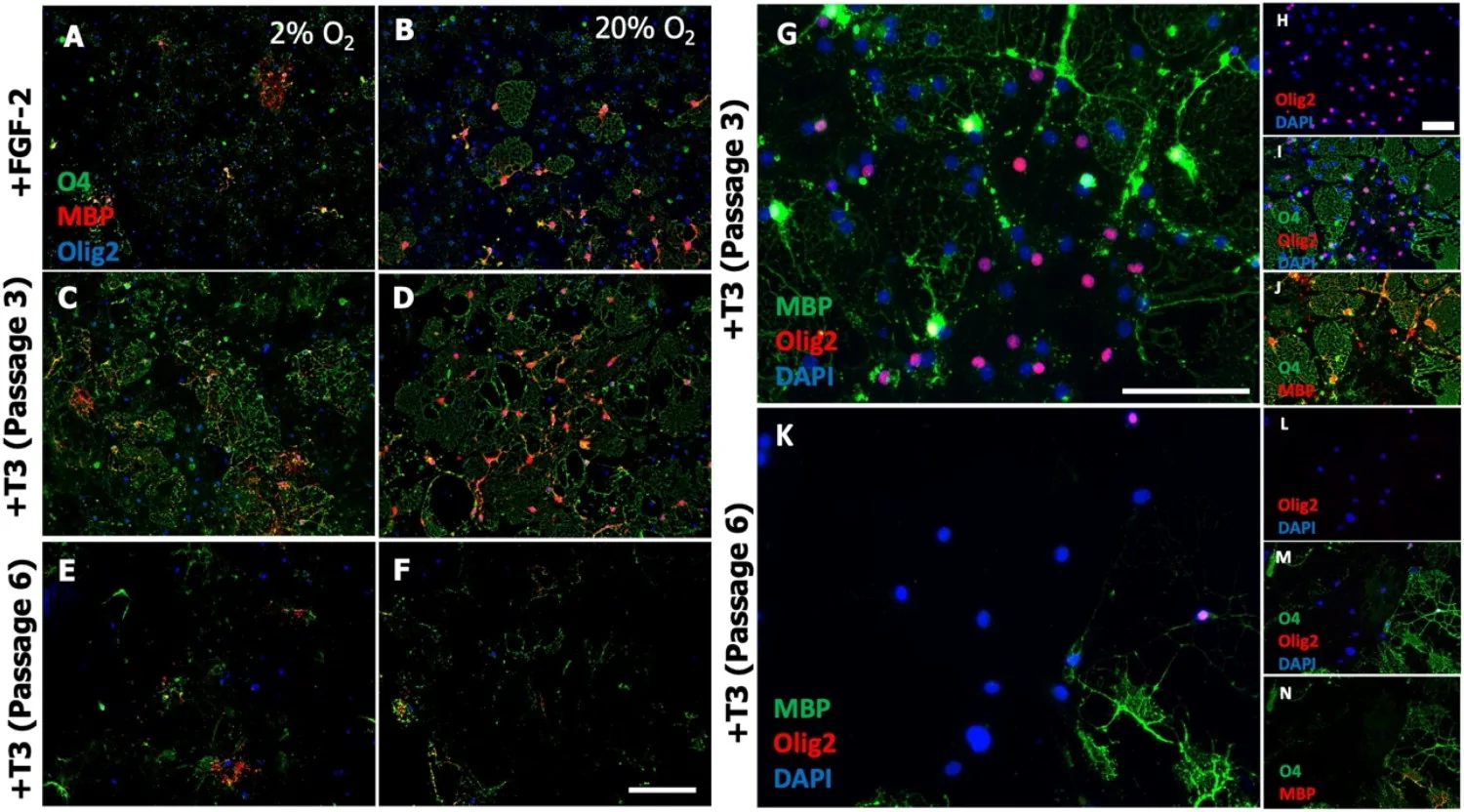
OPCs differentiate into MBP+ mature oligodendrocytes *in vitro* in superphysiological oxygen but most lose their capacity to differentiate after repeated passages. OPCs were grown in B104CM + FGF2 for 3 and 6 passages and then plated onto poly-d-lysine and laminin-coated chamber slides. The growth medium was removed and replaced with B27 growth medium supplemented with 30 nM T3 or 10 ng/mL FGF-2. Cells were maintained in these media for 7 days. Cells were stained for Olig2, MBP and DAPI (Panels A-F,G, K). Cells were stained for O4, Olig2, MBP (Panels H-J and L-N). Scale bar represents 50 µm (A-F) and 100 µm (G,K) and 50 µm (H-J; L-N). Images are representative of three independent experiments.

The presence of B104 CM and FGF-2 was used to maintain the OPCs in a primitive proliferating state. To determine whether these mouse OPCs could mature, OPCs from passage three and passage six were detached and plated onto PDL and laminin-coated chamber slides. They were maintained in FGF-2 for 24 hours to allow them to gradually mature, at which time the medium was replaced with B27 growth medium supplemented with 30 nM T3 or 5 ng/mL FGF-2. The cells were maintained in a 3-gas incubator with 2% O_2_ and with medium changes every 2–3 days for 7 days. Under these conditions, cells expressing Olig2 and MBP were observed in the medium supplemented with T3, but rarely in the medium supplemented with FGF-2 (Figure 6A-D). When the cells were differentiated in an incubator containing 20% oxygen 63 ± 10% of the cells were MBP+ cells by day 7. Many of these cells produced complex sheet-like membranes best visualized with O4 staining (Figure 6G-I). Supplementing the medium with either CNTF, noggin or TGFβ1, or combinations of these factors did not significantly enhance the percentage of cells that were MBP+ (data not shown). When OPCs from passage 6 were differentiated, the majority remained MBP negative (80.8 ± 10%, *p* < 0.5 vs. P3), and were Sox-10 and Olig2 negative (Figure 6E,F,K-N).

## Mouse OPCs Lose Sox-10 Expression and the Capacity to Differentiate between 3 and 6 Serial Passages

Allan et al., (2021) showed that HIF1α formed a transcriptional complex with Olig2 that activated non-canonical HIF targets that blocked OPC maturation by suppressing the expression of the transcription factor Sox10 (Allan et al., 2021).

**Figure 7.**
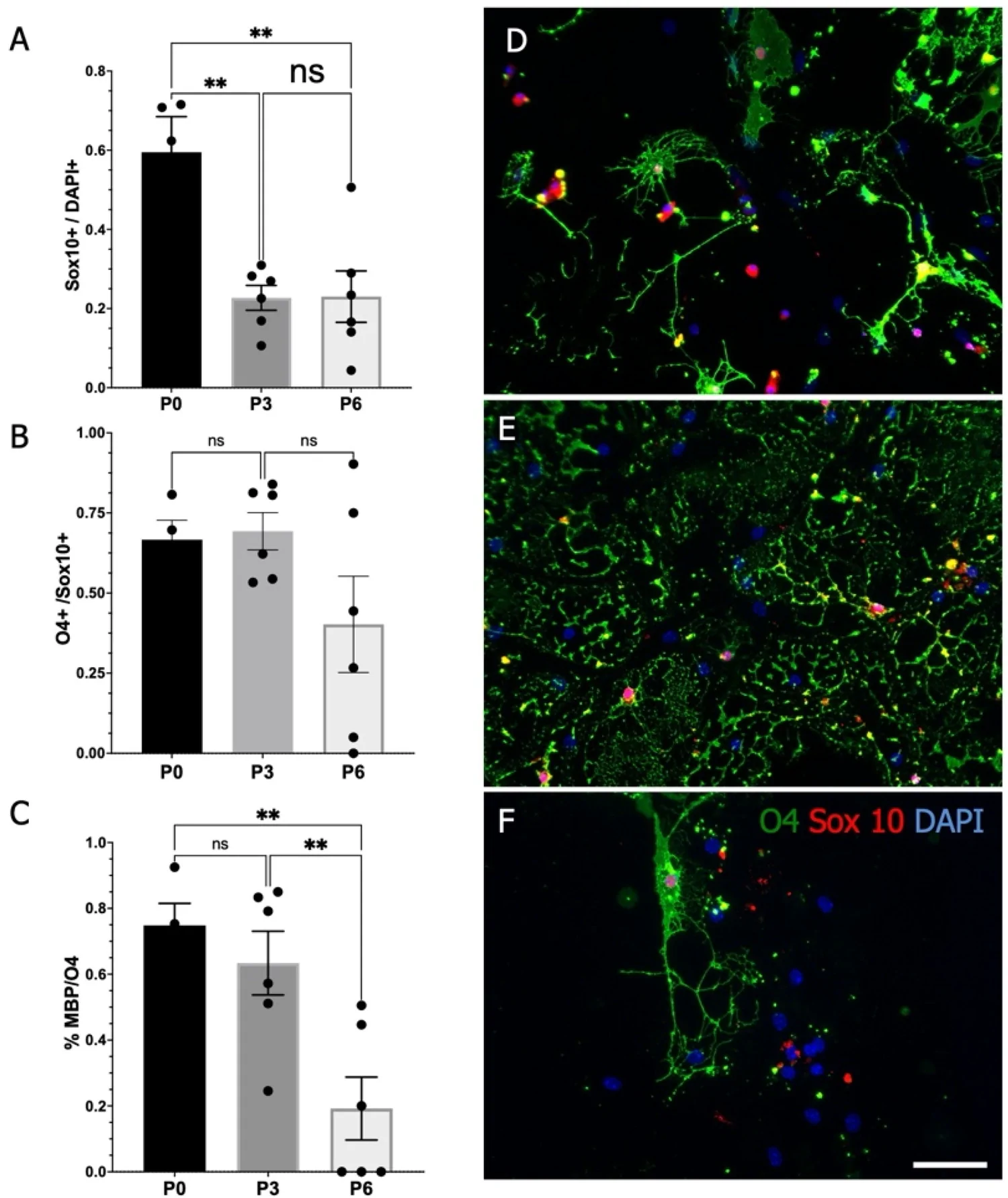
Mouse OPCs lose Sox-10 expression and the capacity to differentiate between 3 and 6 serial passages. OPCs were grown in B104CM + FGF2 for 0, 3 and 6 passages and then plated onto poly-d-lysine and laminin-coated chamber slides. The growth medium was removed and replaced with differentiation medium. Cells were maintained in a 20% O_2_ incubator for 7 days. Cells were stained for O4, Sox10, MBP and DAPI. The percentage of Sox10+ to total DAPI+ cells (A); the percentage of O4+ of total Sox10+ cells (B), and percentage of MBP+ of total O4+ cells (C). Panels E-F are representative images of cells after 7 days of differentiation. Cells depicted are from passages 0, 3 and 6. The magnification bar represents 50 µm. Data are from 1–2 experiments with 4 technical replicates per experiment. **p < 0.01 by one-way ANOVA followed by Tukey’s post hoc test.

Therefore, we suspected that maintaining the OPCs in low oxygen would lead to Sox-10 suppression and that this might explain the failure of the OPCs to differentiate. To test this hypothesis, OPCs were grown in B104CM + FGF-2 for 0, 3 and 6 passages and then plated onto poly-d-lysine and laminin-coated chamber slides. The growth-factor supplemented medium was removed and replaced with differentiation medium. Cells were maintained in a 20% O_2_ incubator for 7 days and then evaluated. Supporting the hypothesis, the percentage of Sox10+ cells decreased between P0 and P3 (60% ± 0.09 at P0 vs. 23% ± 0.03 at P3, *p* < 0.01) but this was not associated with the ability of the cells to acquire O4 or MBP. The percentage of Sox-10+ cells remained at ∼25% at P6, and this was now associated with a decrease in the percentage of cells that had acquired MBP compared to earlier passage cells (63% ± 0.07 at P3 vs. 19% ±0.1 at P6, *p* < 0.01). We did not observe a change in the cellular distribution of Sox-10 from the nucleusto the cytoplasm. A similar reduction in the percentage of Olig2+ cells was seen at P6 (Figure 6). Thus, the continuous propagation of the cells beyond 3 passages (i.e. over approximately 3 weeks in culture) induced changes in key molecular regulator that impaired their capacity to differentiate even when placed into a permissive environment (Figure 7).

## Discussion

Our current knowledge of OPCs has largely been derived from studies on rat OPCs. Rat OPCs from embryonic, neonatal, and adult CNS can be isolated and expanded *in vitro* for more than a year in the presence of PDGF-AA and fibroblast FGF-2 (Barres et al., 1992a; Robinson & Miller, 1996). Yet, relatively few studies have used highly enriched mouse OPC cultures, despite the mouse being the animal of choice for rodent models of demyelinating diseases. Quite simply, it has been technically challenging to obtain highly homogeneous cultures of mouse OPCs *in vitro* in large numbers (Dincman et al., 2012; Medina-Rodríguez et al., 2013; Yoshida et al., 2020). This study establishes a simple method to purify and expand large numbers of mouse OPCs from the brains of neonatal mice *in vitro*.

Initially, we followed the well-established McCarthy and de Vellis protocol to separate the OPCs from microglia, astrocytes and fibroblasts. We observed differences in the number of OPCs produced in the mixed glial cultures that correlated with different lots of FBS. To maximize OPC production during the early stages of the *in vitro* culture, we screened several lots of FBS from different commercial suppliers. The serum chosen for future use was based on the number of OPCs growing on top of the bed layer. A dozen bottles of this lot were purchased and used for all subsequent experiments.

We also compared the proliferation of the mouse OPCs when propagated in PDGF and FGF-2 vs. in B104CM and FGF-2. Bottenstein and her colleagues (1988) showed that rat OPCs proliferated expansively in B104 neuroblastoma conditioned medium; B104CM has been widely used to generate highly enriched primary rat OPC cultures. Subsequent studies have confirmed the presence of PDGF-AA and neuregulin 1 – mitogens that influence OPC growth – in B104CM (Asakura et al., 1997; Canoll et al., 1996; Hu et al., 2012). Mouse OPCs grew faster in B104CM + FGF-2 vs. the PDGF+FGF-2. Previous studies had reported that a greater number of O4+ late-stage OPCs were observed in mouse OPC cultures following isolation from brain tissue than in rat OPC cultures (Horiuchi et al., 2010). This suggested that the initially isolated mouse OPCs were at a more advanced developmental stage and were less proliferative. Therefore, PDGF-AA and FGF-2 would be insufficient to drive their expansion. By contrast, the B104CM, likely due to the neuregulin-1, caused these cells to dedifferentiate to an earlier stage OPC and to proliferate at a greater rate, similar to the effects of EGF reported by Yang et al., 2016. This appears to be a species-specific effect, as this phenomenon is not observed in rat OPC cultures (Yang et al., 2016).

The most significant difference in our method for culturing mouse OPCs was to decrease their exposure to oxygen. Previous studies showed that neural progenitor cell proliferation and differentiation can be affected by growth in 20% vs. 2% oxygen incubators (Moore et al., 2014), and other studies have shown that oxygen tension is a critical determinant in neural stem cell propagation. In those latter studies growth in 5% O_2_ expanded nestin+/CD133+/CD24+ progenitors that readily differentiated into all three neural lineages (Chen et al., 2007a; Pistollato et al., 2007). Therefore, we evaluated the effect of oxygen concentration on mouse OPCs over 6 passages. These studies revealed a major difference in cell proliferation and survival when OPCs were expanded in 2% oxygen. Large cell numbers could be obtained rapidly, and the cells maintained their expression of A2B5/Olig2/Ki67 over several passages. Part of the difference in growth was attributable to reduced cell death, as there were over twice as many apoptotic cells at 96 h in the 20% oxygen condition compared to the 2% oxygen condition, similar to results reported by Yang et al., 2016 (Yang et al., 2016).

An earlier study concluded that cAMP addition was required for mouse OPCs to survive in B104CM + FGF-2 under hyperoxic culture conditions (Horiuchi et al., 2010). However, cAMP addition was not needed in our studies. A plausible hypothesis is that cAMP or downstream targets of protein kinase A counteract the toxic effects of oxidative stress induced by growth in superphysiological oxygen. We did not test this hypothesis. Based on prior studies, we suspect that growing the mouse OPCs in 2% O_2_ induces expression of hypoxia inducible factor, HIF1α, that prevents their maturation. During rodent brain development HIF1α levels are initially relatively high, but then rapidly decrease during the second postnatal week, which is consistent with the role of HIFα as an inhibitor of OPC differentiation. Yuen et al., 2014 demonstrated that culturing OPCs in 2% O_2_ increased both HIF-1α and HIF-2α protein levels. The authors went on to provide direct support for the hypothesis that HIF was inhibiting OPC maturation by elevating HIF1α levels either genetically or using pharmacological agents to prevent the action of its negative regulator Von Hippel–Lindau tumor suppressor. When HIF1α levels were sustained *in vivo* using a genetic approach, OPC differentiation was impaired which lead to hypomyelination (Yuen et al., 2014). Similarly, using a model of chronic HIF1α accumulation in pluripotent-stem-cell-derived OPCs, Allan et al., (2021) showed that HIF1α formed a transcriptional complex with Olig2 that activated non-canonical HIF targets to impair the generation of oligodendrocytes from OPCs. In particular, they showed that HIF1a increased the expression of both Ascl2 and Dlx3, which blocked OPC maturation by suppressing the expression of the transcription factor Sox10 (Allan et al., 2021). Confirming their data, we found that propagating mouse OPCs under conditions where HIF will be stabilized resulted in decreased expression of Sox10.

Multiple approaches have been developed to purify OPCs, including immunopanning and oligosphere formation. Each method has its own benefits and complications. Mouse OPCs can be produced from oligospheres, which involve neurosphere formation from embryonic mouse brains followed by oligosphere induction (Chen et al., 2007b; Pedraza et al., 2008; Zhao et al., 2010). While this method produces oligodendrocytes, it is not clear that they are identical to those formed from postnatal brains since they are derived from stem cells as opposed to primary OPCs. Immunopanning for OPCs has been performed using antibodies to PDGFRα (Macintosh et al., 2023), and OPCs have been enriched from postnatal mouse brains by magnetic cell sorting (Dincman et al., 2012) and 04+ MACS (Flores-Obando et al., 2018), but the yield is very low. Moreover, it has been suggested that these OPCs are limited to two passages before the OPCs differentiate into type 2 astrocytes (Dincman et al., 2012), which is highly impractical for studying these cells.

While we were able to passage the mouse OPCs extensively, they did change with time in culture. More specifically, between 3 and 6 passages they acquired the O4 antigen, as described in rat cultures (Tang et al., 2000), they increased the expression of GLAST and they lost expression of Sox-10. These results are reminiscent of prior work that found that repeatedly passaging early O-2A cells resulted in the transformation of these cells into adult O-2A cells (Wolswijk & Noble, 1989). Other studies have shown that adult O-2As differ from early O-2As in several respects that include cell cycle time, migratory capacity and capacity to differentiate (Raff et al., 1988; Temple & Raff, 1986; Wolswijk & Noble, 1989). Thus, for investigators interested in studying adult O-2As, our method of repeatedly passaging the OPCs will enable them to produce large numbers of cells for analysis relatively simply, whereas acquiring similar numbers of cells directly from the adult brain would be prohibitively complex.

## Conclusion

Our results demonstrate that enriched cultures of mouse brain OPCs can be prepared using a relatively simple and straightforward method. Notably, our results demonstrate that in addition to providing these cells with the appropriate mitogens, mouse OPCs are very sensitive to environmental concentrations of oxygen. To maintain the proliferative capacity of mouse OPCs they must be maintained in a 3-gas incubator that provides 2% O_2_, 5% C0_2_ and 93% N_2_. Under these conditions mouse OPCs will proliferate extensively and when the mitogens are removed the majority of them differentiate into MBP+ oligodendrocytes that have extensive sheet-like membranes; however, beyond 3 passages, they begin to lose their capacity to differentiate into MBP+ oligodendrocytes.

## Data Availability

All data will be made available for inspection or use upon request to the corresponding author.

## Footnotes

1 Since there are differences in glial cell proliferation based on the serum utilized in culture, we prescreened several lots of FBS from Sigma, Atlanta Biological and Life Technologies. Sera selected for use were those that produced the largest numbers of OPCs in the mixed glial cultures with minimal expansion of microglia. Once a lot was chosen, at least 12 bottles were purchased and used for all mixed glial cultures.

## References

[CIT0001] Allan, K. C., Hu, L. R., Scavuzzo, M. A., Morton, A. R., Gevorgyan, A. S., Cohn, E. F., Clayton, B. L. L., Bederman, I. R., Hung, S., Bartels, C. F., Madhavan, M., & Tesar, P. J. (2021). Non-canonical Targets of HIF1a Impair Oligodendrocyte Progenitor Cell Function. *Cell Stem Cell*, *28*(2), 257–272 e211. 10.1016/j.stem.2020.09.019 PMC7867598 33091368

[CIT0002] Althaus, H. H., Montz, H., Neuhoff, V., & Schwartz, P. (1984). Isolation and cultivation of mature oligodendroglial cells. *Die Naturwissenschaften*, *71*(6), 309–315. 10.1007/BF00396614 6472480

[CIT0003] Asakura, K., Hunter, S. F., & Rodriguez, M. (1997). Effects of transforming growth factor-beta and platelet-derived growth factor on oligodendrocyte precursors: insights gained from a neuronal cell line. *Journal of Neurochemistry*, *68*(6), 2281–2290. 10.1046/j.1471-4159.1997.68062281.x 9166720

[CIT0004] Bansal, R., Stefansson, K., & Pfeiffer, S. E. (1992). Proligodendroblast antigen (POA), a developmental antigen expressed by A007/O4-positive oligodendrocyte progenitors prior to the appearance of sulfatide and galactocerebroside. *Journal of Neurochemistry*, *58*(6), 2221–2229. 10.1111/j.1471-4159.1992.tb10967.x 1573402

[CIT0005] Barres, B. A., Hart, I. K., Coles, H. S., Burne, J. F., Voyvodic, J. T., Richardson, W. D., & Raff, M. C. (1992a). Cell death and control of cell survival in the oligodendrocyte lineage. *Cell*, *70*(1), 31–46. 10.1016/0092-8674(92)90531-g 1623522

[CIT0006] Barres, B. A., Hart, I. K., Coles, H. S., Burne, J. F., Voyvodic, J. T., Richardson, W. D., & Raff, M. C. (1992b). Cell death in the oligodendrocyte lineage. *Journal of Neurobiology*, *23*(9), 1221–1230. 10.1002/neu.480230912 1469385

[CIT0007] Behar, T., McMorris, F. A., Novotný, E. A., Barker, J. L., & Dubois-Dalcq, M. (1988). Growth and differentiation properties of O-2A progenitors purified from rat cerebral hemispheres. *Journal of Neuroscience Research*, *21*(2–4), 168–180. 10.1002/jnr.490210209 3216419

[CIT0008] Bögler, O., Wren, D., Barnett, S. C., Land, H., & Noble, M. (1990). Cooperation between two growth factors promotes extended self-renewal and inhibits differentiation of oligodendrocyte-type-2 astrocyte (O-2A) progenitor cells. *Proceedings of the National Academy of Sciences of the United States of America*, *87*(16), 6368–6372. 10.1073/pnas.87.16.6368 PMC54535 2201028

[CIT0009] Bottenstein, J. E., Hunter, S. F., & Seidel, M. (1988). CNS neuronal cell line-derived factors regulate gliogenesis in neonatal rat brain cultures. *Journal of Neuroscience Research*, *20*(3), 291–303. 10.1002/jnr.490200303 2852260

[CIT0010] Canoll, P. D., Musacchio, J. M., Hardy, R., Reynolds, R., Marchionni, M. A., & Salzer, J. L. (1996). GGF/neuregulin is a neuronal signal that promotes the proliferation and survival and inhibits the differentiation of oligodendrocyte progenitors. *Neuron*, *17*(2), 229–243. 10.1016/s0896-6273(00)80155-5 8780647

[CIT0011] Chen, H. L., Pistollato, F., Hoeppner, D. J., Ni, H. T., McKay, R. D., & Panchision, D. M. (2007a). Oxygen tension regulates survival and fate of mouse central nervous system precursors at multiple levels. *Stem Cells (Dayton, Ohio)*, *25*(9), 2291–2301. 10.1634/stemcells.2006-0609 17556599

[CIT0012] Chen, Y., Balasubramaniyan, V., Peng, J., Hurlock, E. C., Tallquist, M., Li, J., & Lu, Q. R. (2007b). Isolation and culture of rat and mouse oligodendrocyte precursor cells. *Nature Protocols*, *2*(5), 1044–1051. 10.1038/nprot.2007.149 17546009

[CIT0013] Dincman, T. A., Beare, J. E., Ohri, S. S., & Whittemore, S. R. (2012). Isolation of cortical mouse oligodendrocyte precursor cells. *Journal of Neuroscience Methods*, *209*(1), 219–226. 10.1016/j.jneumeth.2012.06.017 PMC3407963 22743801

[CIT0014] Dubois-Dalcq, M., Behar, T., Hudson, L., & Lazzarini, R. A. (1986). Emergence of three myelin proteins in oligodendrocytes cultured without neurons. *The Journal of Cell Biology*, *102*(2), 384–392. 10.1083/jcb.102.2.384 PMC2114066 2418030

[CIT0015] Duncan, I. D., Paino, C., Archer, D. R., & Wood, P. M. (1992). Functional capacities of transplanted cell-sorted adult oligodendrocytes. *Developmental Neuroscience*, *14*(2), 114–122. 10.1159/000111655 1396171

[CIT0016] Eisenbarth, G. S., Walsh, F. S., & Nirenberg, M. (1979). Monoclonal antibody to a plasma membrane antigen of neurons. *Proceedings of the National Academy of Sciences of the United States of America*, *76*(10), 4913–4917. 10.1073/pnas.76.10.4913 PMC413048 388422

[CIT0017] Flores-Obando, R. E., Freidin, M. M., & Abrams, C. K. (2018). Rapid and Specific Immunomagnetic Isolation of Mouse Primary Oligodendrocytes. *Journal of Visualized Experiments*, 135, 670. 10.3791/57543 PMC6101296 29863670

[CIT0018] Gard, A. L., Pfeiffer, S. E., & Williams, W. C. (1993). Immunopanning and developmental stage-specific primary culture of oligodendrocyte progenitors (O4^+^GalC^-^) directly from postnatal rodent cerebrum. *Neuroprotocols*, *2*(3), 209–218. 10.1006/ncmn.1993.1027

[CIT0019] Gard, A. L., Williams, W. C., 2nd,., & Burrell, M. R. (1995). Oligodendroblasts distinguished from O-2A glial progenitors by surface phenotype (O4 + GalC-) and response to cytokines using signal transducer LIFR beta. *Developmental Biology*, *167*(2), 596–608. 10.1006/dbio.1995.1051 7875381

[CIT0020] Goldman, J. E., Geier, S. S., & Hirano, M. (1986). Differentiation of astrocytes and oligodendrocytes from germinal matrix cells in primary culture. *The Journal of Neuroscience*, *6*(1), 52–60. 10.1523/JNEUROSCI.06-01-00052.1986 PMC6568630 3511189

[CIT0021] Horiuchi, M., Lindsten, T., Pleasure, D., & Itoh, T. (2010). Differing in vitro survival dependency of mouse and rat NG2+ oligodendroglial progenitor cells. *Journal of Neuroscience Research*, *88*(5), 957–970. 10.1002/jnr.22262 PMC2872551 19908280

[CIT0022] Hu, J.-G., Wu, X.-J., Feng, Y.-F., Xi, G.-M., Wang, Z.-H., Zhou, J.-S., & Lü, H.-Z. (2012). PDGF-AA and bFGF mediate B104CM-induced proliferation of oligodendrocyte precursor cells. *International Journal of Molecular Medicine*, *30*(5), 1113–1118. 10.3892/ijmm.2012.1110 22922759

[CIT0023] Hunter, S. F., & Bottenstein, J. E. (1989). Bipotential glial progenitors are targets of neuronal cell line-derived growth factors. *Brain Research. Developmental Brain Research*, *49*(1), 33–49. 10.1016/0165-3806(89)90057-6 2791266

[CIT0024] Hunter, S. F., & Bottenstein, J. E. (1991). O-2A glial progenitors from mature brain respond to CNS neuronal cell line-derived growth factors. *Journal of Neuroscience Research*, *28*(4), 574–582. 10.1002/jnr.490280415 1870158

[CIT0025] Lafrenaye, A. D., & Fuss, B. (2010). Focal adhesion kinase can play unique and opposing roles in regulating the morphology of differentiating oligodendrocytes. *Journal of Neurochemistry*, *115*(1), 269–282. 10.1111/j.1471-4159.2010.06926.x PMC2939935 20649846

[CIT0026] Li, P., Li, H. X., Jiang, H. Y., Zhu, L., Wu, H. Y., Li, J. T., & Lai, J. H. (2017). Expression of NG2 and platelet-derived growth factor receptor alpha in the developing neonatal rat brain. *Neural Regeneration Research*, *12*(11), 1843–1852. 10.4103/1673-5374.219045 PMC5745838 29239330

[CIT0027] Louis, J. C., Magal, E., Muir, D., Manthorpe, M., & Varon, S. (1992). CG-4, a new bipotential glial cell line from rat brain, is capable of differentiating in vitro into either mature oligodendrocytes or type-2 astrocytes. *Journal of Neuroscience Research*, *31*(1), 193–204. 10.1002/jnr.490310125 1613821

[CIT0028] Macintosh, J., Michell-Robinson, M. A., Chen, X., Chitsaz, D., Kennedy, T. E., & Bernard, G. (2023). An optimized and validated protocol for the purification of PDGFRalpha + oligodendrocyte precursor cells from mouse brain tissue via immunopanning. *MethodsX*, *10*, 102051. 10.1016/j.mex.2023.102051 PMC9939712 36814689

[CIT0029] McCarthy, K. D., & de Vellis, J. (1980). Preparation of separate astroglial and oligodendroglial cultures from rat cerebral tissue. *The Journal of Cell Biology*, *85*(3), 890–902. 10.1083/jcb.85.3.890 PMC2111442 6248568

[CIT0030] Medina-Rodríguez, E. M., Arenzana, F. J., Bribián, A., & de Castro, F. (2013). Protocol to isolate a large amount of functional oligodendrocyte precursor cells from the cerebral cortex of adult mice and humans. *PloS One*, *8*(11), e81620. 10.1371/journal.pone.0081620 PMC3841116 24303061

[CIT0031] Monge, M., Kadiiski, D., Jacque, C. M., & Zalc, B. (1986). Oligodendroglial expression and deposition of four major myelin constituents in the myelin sheath during development. An in vivo study. *Developmental Neuroscience*, *8*(4), 222–235. 10.1159/000112255 2435512

[CIT0032] Moore, L., Bain, J. M., Loh, J. M., & Levison, S. W. (2014). PDGF-responsive progenitors persist in the subventricular zone across the lifespan. *ASN Neuro*, *6*(2), 41. 10.1042/AN20120041 PMC3917572 24367913

[CIT0033] Nishiyama, A. (1998). Glial progenitor cells in normal and pathological states. *The Keio Journal of Medicine*, *47*(4), 205–208. 10.2302/kjm.47.205 9884514

[CIT0034] Nishiyama, A., Komitova, M., Suzuki, R., & Zhu, X. (2009). Polydendrocytes (NG2 cells): Multifunctional cells with lineage plasticity. *Nature Reviews. Neuroscience*, *10*(1), 9–22. 10.1038/nrn2495 19096367

[CIT0035] Noble, M., & Wolswijk, G. (1992). Development and regeneration in the O-2A lineage: Studies in vitro and in vivo. *Journal of Neuroimmunology*, *40*(2–3), 287–293. 10.1016/0165-5728(92)90145-b 1430159

[CIT0036] Noble, M., Murray, K., Stroobant, P., Waterfield, M. D., & Riddle, P. (1988). Platelet-derived growth factor promotes division and motility and inhibits premature differentiation of the oligodendrocyte/type-2 astrocyte progenitor cell. *Nature*, *333*(6173), 560–562. 10.1038/333560a0 3287176

[CIT0037] Pedraza, C. E., Monk, R., Lei, J., Hao, Q., & Macklin, W. B. (2008). Production, characterization, and efficient transfection of highly pure oligodendrocyte precursor cultures from mouse embryonic neural progenitors. *Glia*, *56*(12), 1339–1352. 10.1002/glia.20702 PMC4395472 18512250

[CIT0038] Pfeiffer, S. E., Warrington, A. E., & Bansal, R. (1993). The oligodendrocyte and its many cellular processes. *Trends in Cell Biology*, *3*(6), 191–197. 10.1016/0962-8924(93)90213-k 14731493

[CIT0039] Ping, J., Fu, H., Xiong, Y., Soomro, S., Huang, Z., & Peng, Y. (2022). Poly-L-ornithine blocks the inhibitory effects of fibronectin on oligodendrocyte differentiation and promotes myelin repair. *Neural Regeneration Research*, *18*, 832–839. 10.4103/1673-5374.353493 PMC9700116 36204851

[CIT0040] Pistollato, F., Chen, H. L., Schwartz, P. H., Basso, G., & Panchision, D. M. (2007). Oxygen tension controls the expansion of human CNS precursors and the generation of astrocytes and oligodendrocytes. *Molecular and Cellular Neurosciences*, *35*(3), 424–435. 10.1016/j.mcn.2007.04.003 17498968

[CIT0041] Qin, J., Sikkema, A., Van Der Bij, K., De Jonge, J., Klappe, K., Nies, V., Jonker, J., Kok, J., Hoekstra, D., & Baron, W. (2017). GD1a overcomes inhibition of myelination by fibronectin via activation of protein kinase A: Implications for multiple sclerosis. *The Journal of Neuroscience*, *37*(41), 9925–9938. 10.1523/JNEUROSCI.0103-17.2017 PMC6596593 28899916

[CIT0042] Raff, M. C., Abney, E. R., Cohen, J., Lindsay, R., & Noble, M. (1983). Two types of astrocytes in cultures of developing rat white matter: Differences in morphology, surface gangliosides, and growth characteristics. *The Journal of Neuroscience*, *3*(6), 1289–1300. 10.1523/JNEUROSCI.03-06-01289.1983 PMC6564607 6343560

[CIT0043] Raff, M. C., Lillien, L. E., Richardson, W. D., Burne, J. F., & Noble, M. D. (1988). Platelet-derived growth factor from astrocytes drives the clock that times oligodendrocyte development in culture. *Nature*, *333*(6173), 562–565. 10.1038/333562a0 3287177

[CIT0044] Richardson, W. D., Pringle, N., Mosley, M. J., Westermark, B., & Dubois-Dalcq, M. (1988). A role for platelet-derived growth factor in normal gliogenesis in the central nervous system. *Cell*, *53*(2), 309–319. 10.1016/0092-8674(88)90392-3 2834067

[CIT0045] Robinson, S., & Miller, R. (1996). Environmental enhancement of growth factor-mediated oligodendrocyte precursor proliferation. *Molecular and Cellular Neurosciences*, *8*(1), 38–52. 10.1006/mcne.1996.0042 8923454

[CIT0046] Schnitzer, J., & Schachner, M. (1982). Cell type specificity of a neural cell surface antigen recognized by the monoclonal antibody A2B5. *Cell and Tissue Research*, *224*(3), 625–636. 10.1007/BF00213757 7116415

[CIT0047] Sommer, I., & Schachner, M. (1981). Monoclonal antibodies (O1 to O4) to oligodendrocyte cell surfaces: An immunocytochemical study in the central nervous system. *Developmental Biology*, *83*(2), 311–327. 10.1016/0012-1606(81)90477-2 6786942

[CIT0048] Tang, D. G., Tokumoto, Y. M., & Raff, M. C. (2000). Long-term culture of purified postnatal oligodendrocyte precursor cells. Evidence for an intrinsic maturation program that plays out over months [see comments. *The Journal of Cell Biology*, *148*(5), 971–984. 10.1083/jcb.148.5.971 PMC2174541 10704447

[CIT0049] Temple, S., & Raff, M. C. (1986). Clonal analysis of oligodendrocyte development in culture: Evidence for a developmental clock that counts cell divisions. *Cell*, *44*(5), 773–779. 10.1016/0092-8674(86)90843-3 3948247

[CIT0050] Trotter, J., Karram, K., & Nishiyama, A. (2010). NG2 cells: Properties, progeny and origin. *Brain Research Reviews*, *63*(1–2), 72–82. 10.1016/j.brainresrev.2009.12.006 PMC2862831 20043946

[CIT0051] Trotter, J., Crang, A. J., Schachner, M., & Blakemore, W. F. (1993). Lines of glial precursor cells immortalised with a temperature-sensitive oncogene give rise to astrocytes and oligodendrocytes following transplantation into demyelinated lesions in the central nervous system. *Glia*, *9*(1), 25–40. 10.1002/glia.440090105 8244529

[CIT0052] Velloso, F. J., Kumari, E., Buono, K. D., Frondelli, M. J., & Levison, S. W. (2022). Analyzing mouse neural stem cell and progenitor cell proliferation using EdU incorporation and multicolor flow cytometry. *STAR Protocols*, *3*(1), 101065. 10.1016/j.xpro.2021.101065 PMC8718722 35005647

[CIT0053] Vitry, S., Avellana-Adalid, V., Hardy, R., Lachapelle, F., & Baron-Van Evercooren, A. (1999). Mouse oligospheres: From pre-progenitors to functional oligodendrocytes. *Journal of Neuroscience Research*, *58*(6), 735–751. 10583906

[CIT0054] Wolswijk, G., & Noble, M. (1989). Identification of an adult-specific glial progenitor cell. *Development (Cambridge, England)*, *105*(2), 387–400. 10.1242/dev.105.2.387 2680425

[CIT0055] Yang, J., Cheng, X., Shen, J., Xie, B., Zhao, X., Zhang, Z., Cao, Q., Shen, Y., & Qiu, M. (2016). A Novel Approach for Amplification and Purification of Mouse Oligodendrocyte Progenitor Cells. *Frontiers in Cellular Neuroscience*, *10*, 203. 10.3389/fncel.2016.00203 PMC4992724 27597818

[CIT0056] Yoshida, A., Takashima, K., Shimonaga, T., Kadokura, M., Nagase, S., & Koda, S. (2020). Establishment of a simple one-step method for oligodendrocyte progenitor cell preparation from rodent brains. *Journal of Neuroscience Methods*, *342*, 108798. 10.1016/j.jneumeth.2020.108798 32479973

[CIT0057] Young, G. M., & Levison, S. W. (1997). An improved method for propagating oligodendrocyte progenitors in vitro. *Journal of Neuroscience Methods*, *77*(2), 163–168. 10.1016/s0165-0270(97)00123-4 9489893

[CIT0058] Yuen, T. J., Silbereis, J. C., Griveau, A., Chang, S. M., Daneman, R., Fancy, S. P. J., Zahed, H., Maltepe, E., & Rowitch, D. H. (2014). Oligodendrocyte-encoded HIF function couples postnatal myelination and white matter angiogenesis. *Cell*, *158*(2), 383–396. 10.1016/j.cell.2014.04.052 PMC4149873 25018103

[CIT0059] Zhao, X., He, X., Han, X., Yu, Y., Ye, F., Chen, Y., Hoang, T., Xu, X., Mi, Q. S., Xin, M., Wang, F., Appel, B., & Lu, Q. R. (2010). MicroRNA-mediated control of oligodendrocyte differentiation. *Neuron*, *65*(5), 612–626. 10.1016/j.neuron.2010.02.018 PMC2855245 20223198

[CIT0060] Zhu, X., Bergles, D. E., & Nishiyama, A. (2008). NG2 cells generate both oligodendrocytes and gray matter astrocytes. *Development (Cambridge, England)*, *135*(1), 145–157. 10.1242/dev.004895 18045844

[CIT0061] Zhu, X., Hill, R. A., Dietrich, D., Komitova, M., Suzuki, R., & Nishiyama, A. (2011). Age-dependent fate and lineage restriction of single NG2 cells. *Development (Cambridge, England)*, *138*(4), 745–753. 10.1242/dev.047951 PMC3026417 21266410

